# Analyzing sorbitol biosynthesis using a metabolic network flux model of a lichenized strain of the green microalga *Diplosphaera chodatii*

**DOI:** 10.1128/spectrum.03660-23

**Published:** 2024-12-09

**Authors:** Hadi Nazem-Bokaee, Erik F. Y. Hom, Sarah Mathews, Cécile Gueidan

**Affiliations:** 1 Australian National Herbarium, National Research Collections Australia, NCMI, CSIRO, Canberra, Australia; 2 Synthetic Biology Future Science Platform, CSIRO 261802 , Canberra, Australia; 3 Department of Biology and Center for Biodiversity and Conservation Research, The University of Mississippi, University, Mississippi, USA; 4 Department of Biological Sciences, Louisiana State University, Baton Rouge, Louisiana, USA; 5 Centre for Australian National Biodiversity Research (a joint venture between the Parks Australia and CSIRO) 170489 , Canberra, Australia; University of Porto, Porto, Portugal

**Keywords:** microalgae, systems biology, metabolic network modeling, sorbitol, symbiosis

## Abstract

*Diplosphaera chodatii,* a unicellular terrestrial microalga found either free-living or in association with lichenized fungi, protects itself from desiccation by synthesizing and accumulating low-molecular-weight carbohydrates such as sorbitol. The metabolism of this algal species and the interplay of sorbitol biosynthesis with its growth, light absorption, and carbon dioxide fixation are poorly understood. Here, we used a recently available genome assembly for *D. chodatii* to develop a metabolic flux model and analyze the alga’s metabolic capabilities, particularly, for sorbitol biosynthesis. The model contains 151 genes, 155 metabolites, and 194 unique metabolic reactions participating in 12 core metabolic pathways and five compartments. Both photoautotrophic and mixotrophic growths of *D. chodatii* were supported by the metabolic model. In the presence of glucose, mixotrophy led to higher biomass and sorbitol yields. Additionally, the model predicted increased starch biosynthesis at high light intensities during photoautotrophic growth, an indication that the “overflow hypothesis—stress-driven metabolic flux redistribution” could be applied to *D. chodatii*. Furthermore, the newly developed metabolic model of *D. chodatii*, iDco_core, captures both linear and cyclic electron flow schemes characterized in photosynthetic microorganisms and suggests a possible adaptation to fluctuating water availability during periods of desiccation. This work provides important new insights into the predicted metabolic capabilities of *D. chodatii*, including a potential biotechnological opportunity for industrial sorbitol biosynthesis.

**IMPORTANCE:**

Lichenized green microalgae are vital components for the survival and growth of lichens in extreme environmental conditions. However, little is known about the metabolism and growth characteristics of these algae as individual microbes. This study aims to provide insights into some of the metabolic capabilities of *Diplosphaera chodatii*, a lichenized green microalgae, using a recently assembled and annotated genome of the alga. For that, a metabolic flux model was developed simulating the metabolism of this algal species and allowing for studying the algal growth, light absorption, and carbon dioxide fixation during both photoautotrophic and mixotrophic growth, *in silico*. An important capability of the new metabolic model of *D. chodatii* is capturing both linear and cyclic electron flow mechanisms characterized in several other microalgae. Moreover, the model predicts limits of the metabolic interplay between sorbitol biosynthesis and algal growth, which has potential applications in assisting the design of bio-based sorbitol production processes.

## INTRODUCTION

*Diplosphaera* (Prasiolales, Trebouxiophyceae) is a green algal genus previously attributed to the polyphyletic group of *Stichococcus*-like algae (1), which are cosmopolitan microalgae found in terrestrial, freshwater, and marine environments (2, 3). The type species, *Diplosphaera chodatii*, has been isolated from various substrates, including lichen thalli (2, 4, 5) where it enters a symbiotic relationship with lichenized fungi. More recently, a second species, *D. elongata*, which forms part of the phycobiome of certain lichens, has been described (6, 7). *Diplosphaera chodatii* was shown to have high desiccation tolerance due to its ability to synthesize low molecular weight carbohydrates that act as protective compounds, such as sorbitol (8). In the class of green microalgae Trebouxiophyceae, sorbitol has also been reported as a potential osmolyte enabling different algae to withstand high salinity stress (9). *D. chodatii* has been identified as the photobiont of *Endocarpon pusillum,* in addition to other lichens (2, 10). Based on ^14^C experiments and transcriptome data, it is thought that *D. chodatii* synthesizes and transfers sorbitol to its fungal partner, *E. pusillum*, during the lichen symbiosis (11, 12).

Sorbitol is a six-carbon sugar alcohol with numerous industrial applications. The U.S. Department of Energy listed sorbitol as one of the top ten platform chemicals, which act as a precursor to producing various other higher value-added products (13). Sorbitol has already been manufactured at large-scale for both direct applications (e.g., as an ingredient of toothpaste) and as an intermediate in the production of vitamin C, Sorbitan (a surfactant), and Isosorbide, the precursor of many polymers such as polyester (14, 15). It could also be used potentially as an intermediate to produce alkanes and other hydrocarbons (13, 16). Sorbitol is mainly produced by the chemical hydrogenation of glucose (15). High reaction temperatures and hydrogen requirements, which are major drawbacks of the well-established chemical sorbitol production, have led to considerations of less-energy-intensive bio-based production processes (17). Biosynthetic processes for sorbitol production have focused primarily on using the facultative anaerobic bacterium *Zymomonas mobilis* (18) although other microorganisms, including *Lactobacillus plantarum* and *Saccharomyces cerevisiae,* have also been considered (14, 19). Increasing global demands for sorbitol (20) as well as the high selectivity and specificity of biosynthetic sorbitol processes (versus chemical synthesis) are potential drivers for the development of efficient biotechnological processes for sorbitol production in the future.

Although *D. chodatii* was isolated more than four decades ago, a comprehensive understanding of the metabolic capabilities of *D. chodatii* is still lacking. While photoautotrophic growth—the utilization of light energy to fix atmospheric carbon dioxide—has been reported for free-living *D. chodatii* (8), there have been no reports of the possibility of heterotrophic growth (utilization of only organic carbon) or mixotrophic growth (utilization of both organic and inorganic carbon) for this species to date as far as we are aware. However, both heterotrophic and mixotrophic growth have been reported within the Trebouxiophyceae (21–23). Previous studies showed that mixotrophic ability boosts the growth and biomass formation of several green microalgae (24). Mixotrophy not only enhances biomass production, it can also lead to the production of invaluable biomolecules such as polysaccharides, lipids, and vitamins. For example, *Chlorella vulgaris* grown with glucose and carbon dioxide showed a twofold increase in its ability to produce exopolysaccharides compared with growth on carbon dioxide alone (25). A better understanding of *D. chodatii* growth at the molecular level will inform potential strategies for improved sorbitol biosynthesis by this alga. The potential of using metabolic network flux models to predict metabolic capabilities and advance the understanding of lichenized fungi and their algal symbionts, such as *D. chodatii,* has previously been highlighted (26).

Through a careful inventory of enzyme-catalyzed reactions representing various metabolic pathways, metabolic flux models can reveal the subcellular functions of a specific microorganism (27). Such reactions can be translated into a set of mathematical equations that can be optimized using condition-based constraints to provide realizable solutions to predefined problems. For example, such metabolic reconstructions can be used to maximize the cellular growth rate or the secretion rate of a specific metabolite. Flux balance analysis (FBA) is one of the most common techniques (28) used to solve this metabolic system of equations through either minimization or maximization of an “objective function” subject to a set of constraints defined implicitly by the system conditions (e.g., growth media composition) or that are imposed on the system (e.g., measured metabolite fluxes) (29–31). So-called “constraint-based modeling” of metabolism enables a wide range of applications including but not limited to informing metabolic engineering strategies and identifying genetic mutants (32–34), drug targeting and production of chemicals (35), and creating databases/knowledgebases of biochemical and functional gene information (36, 37). It can also be used to identify optimal strains and culture media conditions (38, 39) and, therefore, improve the growth of organisms difficult to culture *in vitro*.

We describe our efforts to develop a computational framework to explore and predict physiological aspects of *D. chodatii* growth (i.e., autotrophic versus mixotrophic growth) in order to gain a better understanding of sorbitol biosynthesis by the alga in the free-living state. The long-term aim of the present study is to apply the metabolic modeling framework of *D. chodatii* to explore its potential as a platform for biotechnological sorbitol production. Furthermore, the metabolic model of *D. chodatii* can be combined with the metabolic models of their respective fungal symbionts (as they emerge) to facilitate the study of metabolic interplays between symbionts, which are extremely difficult to culture and study *in vitro*. Recently, the full genome of a strain of *D. chodatii* isolated from *E. pusillum* was assembled and annotated (10). Here, we reconstructed the core metabolic network of *D. chodatii* using this algal genome. Using a constraint-based modeling approach, a flux-balanced profile of the core metabolic assemblage of *D. chodatii* was obtained to inform substrate and energy requirements for growth and sorbitol biosynthesis under different culturing conditions.

## MATERIALS AND METHODS

### Development of the core metabolic model of *Diplosphaera chodatii*

#### Step 1: collecting information on protein-encoding genes with metabolic functions

The objective of this step was to identify and extract protein sequences with metabolic functions from the genome of *D. chodatii* CS-1475 (ANACC, CSIRO, Hobart, Australia). Protein sequences were obtained from the recently published genome of *D. chodatii* CS-1475 (PRJNA606981; JAALGY000000000; Files S1 and S2) (10). These protein sequences were mapped to identify gene ontologies and annotated to assign reliable protein function labels using the OmicsBox annotation pipeline version 2.1.14 (40), as described previously (10). The Combined Pathway Analysis feature of OmicsBox was used as part of the functional analysis to inform protein sequences linked to the Kyoto Encyclopedia of Genes and Genomes (KEGG) pathways (41). The resulting functional annotated protein sequences can be found in File S5 along with their corresponding sequence, Gene Ontology (GO), an enzyme information. Before drafting the metabolic network reconstruction (step 2 below), this list of functionally annotated proteins was filtered to remove any sequence description containing one of the following text strings: "predicted protein," "hypothetical protein," "putative," "unnamed," "transcription," "translation," "DNA," "RNA," "ribosomal," "ribosome," "helicase," "ribonuclease," "movement," or “signaling.” At this stage, most remaining protein sequences were those that were assigned to one or more EC number classes (either complete or incomplete EC numbers). Finally, the NCBI BLASTp suite was used to determine if the remaining protein sequences that were not assigned to any EC number class matched with a BLAST hit with known enzymatic functions.

#### Step 2: reconstructing a draft metabolic network

The EC numbers collected in step 1 were searched against the KEGG (41), BiGG (37), and MetaCyc (42) databases to extract enzymatic reactions and metabolic pathways associated with each identified protein function. Data on metabolites (e.g., charge, chemical formula) were also extracted from these databases. The metabolic reconstruction followed the BiGG convention for identifiers of reactions and metabolites (37). A list of 2,232 metabolic reactions, comprising 1,541 metabolites across 14 compartments, was created for further processing. Transport reactions were added for metabolite translocation across different compartments. Additionally, reactions were added to the reconstruction to enable metabolite exchange with the extra-cellular environment, i.e., assimilation/secretion of metabolites across the cell. In formulating the biomass equation of the metabolic network and to account for cell growth and growth-associated ATP requirements, it was assumed that one gram of biomass (i.e., dry cell weight, DCW) is produced from key precursor metabolites, ATP, and essential cofactors with stoichiometric ratios identical to those of *Synechocystis* sp. PCC 6803 (43). The key precursor metabolites in the biomass equation are glucose 6-phosphate, erythrose 4-phosphate, ribose 5-phosphate, glyceraldehyde 3-phosphate, pyruvate, phosphoenolpyruvate, 3-phosphoglycerate, oxaloacetate, and acetyl-CoA (File S3). The ATP requirement value for growth-associated maintenance (GAM) was set to 53.35 and 38.89 mmol/gDCW for autotrophic and mixotrophic growth, respectively. These estimated GAM values were initially calculated based on the elemental composition measurements of the cell cultures of the cyanobacteria *Synechocystis* sp. PCC 6803 (43). GAM values were available through the biomass equation of the iRC1080 metabolic model of eukaryotic alga *Chlamydomonas reinhardtii*, but these values were initially calculated using *Escherichia coli* elemental composition measurements (44). For this reason, we preferred to use the GAM values of *Synechocystis* sp. PCC 6803. The value of non-growth associated maintenance of ATP was based on that for *C. reinhardtii*: 2.85 mmol/gDCW.h (45).

#### Step 3: filling gaps and manual refinement of the metabolic network reconstruction

A series of automated and manual refinement steps were applied to improve the connectivity and quality of the metabolic network in the draft reconstruction generated in step 2. First, only reactions participating in central carbon and energy metabolism were retained, which included glycolysis/gluconeogenesis, pyruvate metabolism, oxidative phosphorylation, the tricarboxylic acid cycle (TCA) and Calvin-Benson cycles, and the sugar metabolism and pentose phosphate pathways. Predictions of metabolic fluxes from core metabolic models have been shown to be comparable to those from their genome-scale counterparts for various microorganisms, while they shorten the process of gap filling and allow the use of simplified biomass equations (46–48).

Although tools for automating metabolic reconstruction of algal genomes have become available (such as the Rapid Annotation of Photosynthetic Systems [49]), we focused on manual reconstruction and gap-filling of *D. chodatii* metabolism for the following reasons: first, metabolic reconstructions generated through automated pipelines still require thorough gap-filling and manual refinement to create useful metabolic models, and existing automated gap-filling procedures are still immature and pose negative impacts on metabolic reconstructions, particularly for un-conventional microorganisms such as *D. chodatii*; second, most existing automated pipelines extract information from resources that are heavily supplemented with data from unrelated microbial systems, rendering them less relevant to green algae including *D. chodatii*; third, almost all of the automated reconstruction approaches rely, as the reference, on model algal genome scale metabolic network reconstructions such as that for *C. reinhardtii*.

All reactions were checked for compartmental assignments using the list of functional protein annotations and published literature to fix or remove incorrectly allocated reactions. For example, in eukaryotes, NAD-dependent isocitrate dehydrogenase (EC: 1.1.1.41) occurs only in mitochondria (50); however, during the reconstruction process, two identical reactions were assigned to this enzyme, one in the mitochondrion and the other in the cytoplasm, so only the mitochondrial copy was kept. All enzymes in the reconstructed metabolic network were subsequently checked for the correct assignment of isozymes and enzyme complexes. For instance, the oxoglutarate dehydrogenase complex consists of several sub-units: a 2-oxoglutarate decarboxylase (EC: 1.2.4.2), a dihydrolipoamide succinyl-transferase (EC: 2.3.1.61), and a dihydrolipoamide dehydrogenase (EC: 1.8.1.4) (51). Therefore, a single lumped reaction representing the multi-unit oxoglutarate dehydrogenase complex was kept preventing the addition of intermediate dead-end metabolites in the core metabolic network. In this step, the gene-protein-reaction associations were also created and checked manually.

#### Step 4: reconstructing ATP generation and electron flow in the mitochondrion

The annotated genome of *D. chodatii* encodes for all enzymes of the mitochondrial oxidative phosphorylation pathway (i.e., electron transport chain or ETC). The objective function was chosenThe objective function was chosenReconstruction of the metabolic network of *D. chodatii* follows the accepted stoichiometric ratios of electrons, protons, and ATPs for the ETC of a model eukaryotic cell, as described below, as these ratios have not yet been measured for *D. chodatii*. Reduced NAD generated via the TCA cycle in the mitochondrion initiates electron flow in the ETC by passing electrons to a quinone in the H^+^-translocating NADH-ubiquinone dehydrogenase (i.e., complex I). It is generally agreed that the ratio of protons to electrons translocated by complex I is 3 protons transferred to the cytosol for every 2 electrons coming from NADH (52–55). The second enzyme complex in ETC, succinate dehydrogenase, passes another 2 electrons from mitochondrial succinate to ubiquinone. Reduced ubiquinone, deriving from complex I and II, is oxidized through ubiquinol-cytochrome *c* oxidoreductase (i.e., complex III), by passing 4 electrons to cytochrome *c* and releasing another 4 protons into the cytosol. Reduced cytochrome *c* passes electrons to oxygen as the terminal electron acceptor in cytochrome *c* oxidoreductase (i.e., complex IV), which pumps 2 more protons out of the mitochondrion to the cytoplasm (56). The resulting proton gradient (9 H^+^ on the cytosolic side of the membrane) drives ATP synthesis by the combined action of an F-type ATP synthase (pumping 8 protons into mitochondrial space) and a phosphate carrier, transporting 1 of the cytosolic protons together with phosphate. The stoichiometric ratio of protons to 1 molecule of ATP (H^+^/ATP) has been determined to be 2.67 based on the established structural information on the c-ring of ATP synthase of animal mitochondria (54, 57). ATP molecules generated in mitochondria are carried by an ADP/ATP carrier to the cytoplasm to provide energy for algal glycolysis and biomass production. Therefore, the overall stoichiometry (per mole of oxygen) of ETC in mitochondria can be shown as in equation 1 involving the net transfer of 4 electrons:


(1) $$O_{2}+2\;NADH+20\;H_{mitochondrion}^{+}\to 2\;H_{2}O+2\;NAD^{+}+\;18\;H_{cytosol}^{+}$$

#### Step 5: reconstructing light-dependent electron flow and ATP generation in chloroplast

In the absence of species-specific data for *D. chodatii*, reaction stoichiometries and electron pathways in model green microalgae (i.e., *Chlamydomonas reinhardtii*) or model unicellular cyanobacteria (i.e., *Synechocystis* sp. PCC 6803) were used to represent photosynthesis by the *D. chodatii* metabolic network reconstruction as explained below. Electrons released by the split of water molecules, energized by light in the chloroplast of *D. chodatii*, reduce plastoquinone in photosystem II (i.e., PSII). It was assumed that both linear electron flow (LEF) and cyclic electron flow (CEF) mechanisms identified for photosynthetic organisms (58, 59) exist in *D. chodatii*. Briefly, the LEF mechanism channels all electrons coming from PSII into plastocyanin, which in turn reduces a ferredoxin in photosystem I (i.e., PSI) activated by light. In contrast, the CEF mechanism only passes half of the electrons in plastoquinone to plastocyanin, while the remaining electrons drive translocation of protons from the stroma to the thylakoid by a cytochrome b_6_/f oxidase. Thus, the CEF steepens the proton gradient across the lumen of the chloroplast. Electron flow is completed by a ferredoxin-NADP^+^ reductase, which reduces NADP by regenerating the ferredoxin required for electron shuttling in PSI. The overall stoichiometry of water oxidation by light reactions in *D. chodatii* can be shown in equation 2:


(2) $$2\;H_{2}O+8\;hv+2\;NADP^{+}+10\;H_{stroma}^{+}\to O_{2}+2\;NADPH+\;12\;H_{thylakoid}^{+}$$

Here, *hv* represents photons that provide energy for splitting water molecules.

#### Step 6: converting metabolic reconstruction to a computable format

The reconstructed metabolic network of *D. chodatii* was converted from textual format into a COBRA-compatible model (60) within MATLAB (www.mathworks.com) for further gap-filling and flux analysis. All reactions were checked and corrected for mass and charge balance. The directionality of all reactions was checked using thermodynamic data available in MetaCyc (42). The refined metabolic model of *D. chodatii* was named iDco_core according to the existing conventions (61). This model is available in Excel format (File S4).

#### Step 7: simulating metabolism and predicting metabolic fluxes

FBA (28) optimization problems were solved using both the GNU Linear Programming Kit solver (http://www.gnu.org/software/glpk/) and the built-in MATLAB solver in MATLAB with the COBRA toolbox (60). FBA was performed by solving the following Linear Programming (LP) problem:


$$\begin{array}{cc} Maximize & biomass/sorbitol \end{array}$$

(1) $$\begin{array}{cc} \begin{array}{cc} subject\;\;to & \sum_{j}S_{ij}v_{j}=0, \end{array} & \begin{array}{cc} \forall i\in I,\;j\in J & \end{array} \end{array}$$

(2) $$\;\begin{array}{cc} LB_{j}\leq v_{j}\leq UB_{j} & \forall j\in J \end{array}$$

(3) $$\begin{array}{cc} v_{j}\in R & \forall j\in J \end{array}$$

where sets, variables, and parameters are defined as follows:

Sets:

- $I=\left\{i|i=1,2,\cdots ,M\right\}$ = set of metabolites in the stoichiometric model.
- $J=\left\{j|j=1,2,\cdots ,N\right\}$ *=* set of reactions in the stoichiometric model.

Variables:

- *v _j_ =* flux of reaction *j*∈*J*.

Parameters:

- *S_ij_ =* stoichiometric coefficient of metabolite *i*∈*I* in reaction *j*∈*J*.
- *UB_j_ =* upper bound for the flux of reaction *j*∈*J*.
- *LB_j_ =* lower bound for the flux of reaction *j*∈*J*.

The objective function was chosen to be either algal growth (maximizing flux through the biomass reactions; see File S3) or sorbitol production (maximizing flux through sorbitol exchange). All reaction fluxes in the models are in mmol/gDCW/h except for the reaction representing cell biomass formation, which is expressed as h^−1^. The bounds for water, proton, phosphate, and oxygen were left unconstrained for all simulations. For simulating autotrophic growth, both the lower and upper bounds of carbon dioxide exchange were set to 10 mmol/gDCW/h, while the lower bound of glucose demand reaction was constrained to zero (no uptake). For simulating the mixotrophic growth, glucose uptake was fixed by constraining the lower and upper bounds of glucose demand reaction to one mmol/gDCW/h, while carbon dioxide uptake was left unconstrained. The lower and upper bounds of photon exchange were constrained to −1,000 and zero, respectively, to allow for only photon uptake in all autotrophic and mixotrophic growth simulations. For converting photon flux from mmol/gDCW/h to µmol/m^2^/s, it was assumed that the surface area per kilogram biomass for *D. chodatii* cells is 389 m^2^/kg, similar to that calculated for *C. reinhardtii* (44). Geometric flux balance analysis was also performed in addition to FBA to identify the optimal single unique solution representative of the space of all possible fluxes (62). Flux variability analysis was performed to obtain a range of possible fluxes under optimal growth conditions as described previously by Mahadevan and Schilling (63).

## RESULTS AND DISCUSSION

### Properties of the metabolic model of *D. chodatii*

#### Reconstructed metabolic pathways and compartments

Starting from the assembled genome of *D. chodatii* CS-1475 functions for 21,261 proteins were predicted (see File S5 for the full list). Eleven thousand one hundred ninety-seven of these protein sequences were annotated with GO terms, and 6,108 of these annotations included an Enzyme Commission (EC) number. Of these, only 1,951 protein sequences included full 4-level EC numbers (e.g., EC:4.2.1.13 or fructose-bisphosphate aldolase). These protein sequences were mapped to 1,135 enzyme-catalyzed reaction entries, spanning 63 metabolic pathways and 14 compartments, forming the draft reconstruction of the core metabolic network. At this stage, two identical chemical reactions occurring in different compartments were considered as two separate reaction entries. These reaction entries underwent an extensive automated and manual refinement process (step 3 of Materials and Methods), and 194 unique metabolic reactions were retained, that are involved in 12 core metabolic pathways and five compartments (Table 1). The current version of the iDco_core metabolic model of *D. chodatii* also contains 155 metabolites and 151 genes (Table 1). Among the removed reactions, 114 reactions are involved in nucleotide biosynthesis, 185 in lipid and fatty acid biosynthesis and degradation, 244 in amino acid biosynthesis and degradation, and 366 in other metabolic pathways such as cofactor and amino sugar biosynthesis pathways. Additionally, 32 of the removed reactions were those assigned to a compartment where processes do not contribute to core metabolism. This included five reactions of the glycolysis and pyruvate metabolic pathways that occur in the flagella and 27 reactions that occur in the periplasm.

**TABLE 1:** Statistics on the core metabolic model reconstructed for *Diplosphaera chodatii,* iDco_core

Properties of the iDco_core metabolic model of *Diplosphaera chodatii*		
Cellular compartments	Number of metabolites	Percent of total
Cytosol	53	34.2%
Mitochondrion	38	24.5%
Thylakoid	6	3.9%
Chloroplast	47	30.3%
Extracellular	11	7.1%
Total	155	100.0%

Metabolic pathways	Number of reactions	Percent of total
Biomass	2	1.0%
Reductive pentose phosphate (CBB^*a*^) cycle	8	4.1%
Tricarboxylic acid (Krebs) cycle	19	9.8%
Energy maintenance	1	0.5%
Exchange	14	7.2%
Glycolysis/gluconeogenesis	25	12.9%
Oxidative phosphorylation	8	4.1%
Oxidative pentose phosphate pathway	9	4.6%
Pentose phosphate pathway (other)	9	4.6%
Photosynthesis	6	3.1%
Pyruvate metabolism	27	13.9%
Starch and sucrose metabolism	3	1.5%
Transport	53	27.3%
Other	10	5.2%
Total	194	100.0%

^*a*^ Calvin-Benson-Bassham.

#### *Predicted protein transporters in* D. chodatii

The annotated genome of *D. chodatii* contains over 506 protein sequences with functions belonging to the main families of protein transporters and carriers (Files S5 and S6). It accounts for over 69 major facilitator superfamily transporters, 124 ATP-binding cassette family of transporters, and 21 carbohydrate and sugar transport functions. Over 145 protein sequences are annotated as carrier proteins in the genome of *D. chodatii* (File S5). Among these carrier proteins, there are dicarboxylate (e.g., malate) transporters, and ADP/ATP carrier proteins in both mitochondria and chloroplasts (File S6). A simple diffusion mechanism was assumed for sorbitol transport across compartments since the annotated genome of *D. chodatii* does not account for a specific transport family for sorbitol. Transporter proteins listed in File S6 were included in the model.

### *In silico* analyses of metabolic capabilities of *D. chodatii*

#### Analysis of growth predicted by the iDco_core metabolic model

**Fig 1:**
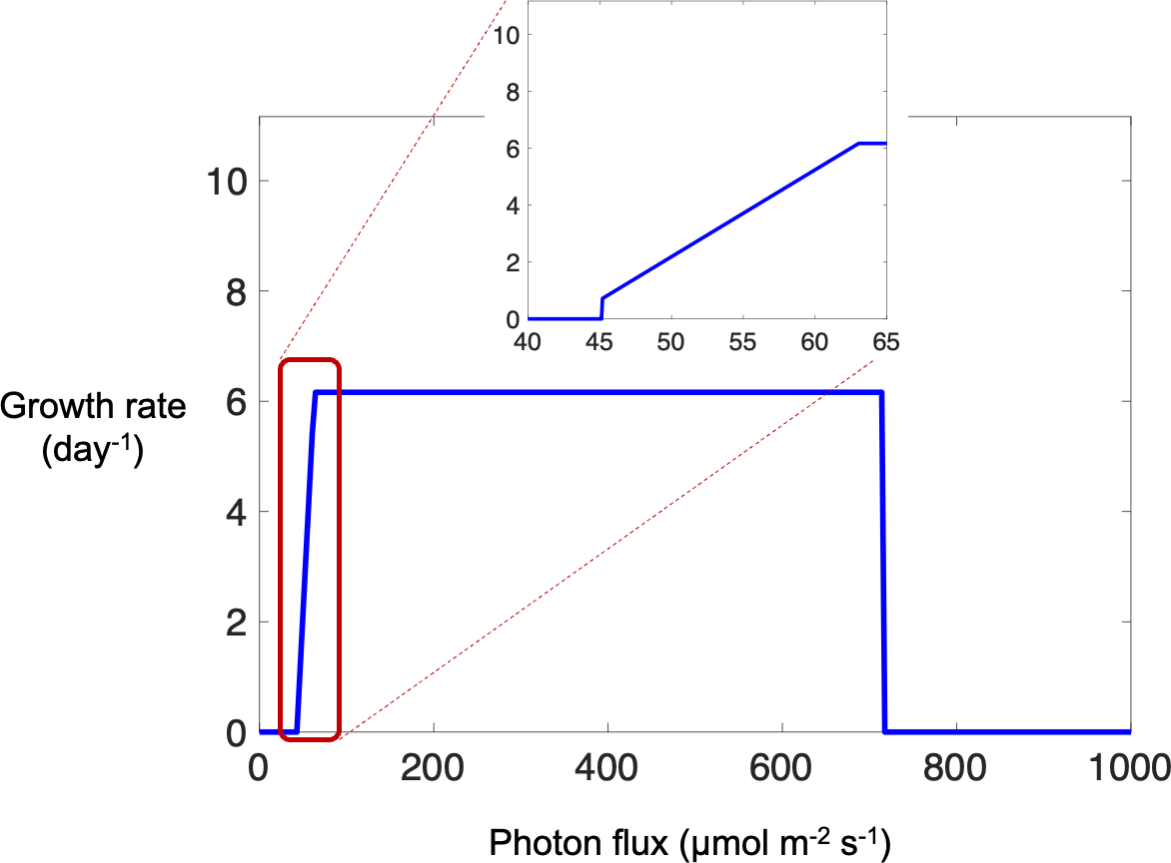
Photoautotrophic growth (per day) of *D. chodatii* simulated by the iDco_core metabolic model at different light intensities shown as a function of varying photon fluxes from 0 to 1000 µE/m^2^/s. No feasible solutions (i.e., no growth) were predicted by the model at photon fluxes below 45 or above 714 µE/m^2^/s. Carbon dioxide was the sole carbon source for all simulations shown. The inset (red box) shows expanded details.

The iDco_core metabolic model of *D. chodatii* predicts photosynthetic growth *in silico* for a range of photon fluxes from 45.2 µmol/m^2^/s (low light) to 714 µmol/m^2^/s (high light) (Fig. 1). The predictions of the model align with an experimental study that measured the lowest growth rate by *D. chodatii* at photon flux of 35 µmol/m^2^/s and showed that cultures of *D. chodatii* reached photo-bleaching at 846 µmol/m^2^/s (8). The maximum theoretical biomass yield predicted by the iDco_core model is 0.0257 g dry cell weight per mmol of fixed carbon dioxide (as the sole carbon source), which is equal to a growth doubling rate of ~6.2 day^−1^ (Fig. 1). This is in agreement with the observation of the maximum growth rate that was reached after 5 days of growth under optimum growth conditions (8). At photon fluxes above 714 µmol/m^2^/s, no growth was predicted by the iDco_core model, but high metabolic flux for cycling was predicted between starch biosynthesis and degradation pathways. Carbon storage in chlorophytes occurs via the synthesis and storage of starch in chloroplasts (64). The elevated starch biosynthesis/degradation predicted by the model is due to the fixation of excess carbon in the Calvin-Benson-Bassham (CBB) cycle to dissipate additional electrons coming into the metabolic network in response to high photon flux. It is likely that the “overflow hypothesis” plays a role under high light intensity during the autotrophic growth of *D. chodatii*. The overflow hypothesis, initially described as overflow metabolism, was observed by Neijssel and Tempest for heterotrophic microbes (65), and according to this hypothesis, stress (nutrient deprivation, high light intensity, etc.) results in the diversion of carbon flux from cellular growth to starch and/or oil biosynthesis. This phenomenon has been also observed in both microalgae (66–68) and higher plants (69, 70). A potential technological strategy for producing sorbitol might focus on growing *D. chodatii* autotrophically at high light intensities to achieve high levels of starch biosynthesis for subsequent conversion to sorbitol.

To examine whether mixotrophic growth by *D. chodatii* is supported *in silico*, the iDco_core model was run in the presence of both glucose and carbon dioxide. The choice of glucose was based on the presence of four genes in the genome of *D. chodatii* annotated as a plastidic glucose transporter (File S5). For simulating the mixotrophic growth of *D. chodatii*, flux through the mixotrophic biomass reaction was maximized at varying levels of photon flux up to 714 µmol/m^2^/s. The maximum theoretical biomass yield was predicted at 0.028 g dry cell weight per total mmoles of substrates (i.e., glucose and carbon dioxide). The maximum biomass yield predicted by the model for mixotrophic growth is higher than that for autotrophic growth because additional carbon dioxide is fixed as photon flux increases. This is in line with the previous observations of increased biomass for several microalgae during mixotrophic growth (24). During the mixotrophic growth of *D. chodatii,* our model predicts that the oxidative pentose phosphate and partial reductive pentose phosphate (CBB cycle) pathways are active, whereas, in autotrophic growth, only the complete CBB cycle is active. A similar flux distribution has been reported for *Synechocystis* sp. PCC 6803 (43). Our model predicts that an increase in photon fluxes results in an increase in flux through phosphogluconate dehydrogenase (EC: 1.1.1.44) and phosphoenolpyruvate carboxykinase (EC: 4.1.1.49) to regenerate NADPH and assimilate carbon dioxide, respectively, in the cytosol. It should be noted that glucose uptake does not occur through glycolysis during the mixotrophic growth, according to the model predictions. Instead, glucose is utilized by the D-Glucitol:NADP 1-oxidoreductase (reactions SBTR and SBTRh in the model) to reduce it to sorbitol (File S4).

Table 2 shows the properties of the metabolic network reconstructions for *Synechocystic* sp. PCC6803 and *Chlamydomonas reinhardtii*, and *Chlorella vulgaris* UTEX 395 as compared with the iDco_core reconstruction for *D. chodatii*. Based on the results shown in Table 2, iDco_core metabolic reconstruction is more of an algae/cyanobacterium hybrid reconstruction; however, the iDco_core model enables simulating (i) sorbitol biosynthesis, (ii) exchange of metabolites across different compartments using special carriers (e.g., ATP/ADP carrier protein) and transporters (e.g., dicarboxylate antiporters), which have not been captured by the previous models listed in Table 2.

**TABLE 2:** Properties of metabolic network reconstructions for selected cyanobacteria and green microalgae compared with the iDco_core metabolic reconstruction for *Diplosphaera chodatii*

	*Synechocystic* sp. PCC6803	*Chlamydomonas reinhardtii*	*Chlorella vulgaris* UTEX 395	*Diplosphaera chodatii*
Taxonomy	Cyanobacteria	Chlorophyta	Chlorophyta	Lichenized Chlorophyta
Genome size (Mb)	3.57	100	63	85.6
Scale of metabolic model reconstruction	Core	Genomic	Genomic	Core
Metabolic model ID	–^*a*^	iRC1080	iCZ843	iDco_core
Reference	(43)	(45)	(71)	This study
Number of genes	–^*a*^	1,073	843	151
Number of reactions	70	1,985	2,286	194
Number of metabolites	46	1,825	1,770	155
Number of compartments	–^*a*^	5	6	5
Predicted biomass yield (gDCW/C-mol)^*b*^	24.3/26.3	13.98/−	1.92/2.72	25.7/28.1

^*a*^ “–”, signifies no ID and the number of genes and compartments for this model were not reported.

^*b*^ Photoautotrophic growth/Mixotrophic growth.

Green microalgae synthesize ATP in both mitochondria and chloroplasts. Flux analysis by the iDco_core metabolic model supports ATP synthesis in both compartments and ATP/ADP carriers are active in both organellar compartments to allow the transfer of ATP across compartments. Additionally, the iDco_core model supports electron flow and proton displacement through established linear and cyclic electron flow pathways, details of which are described in the following section.

#### The interplay between electron flow mechanisms and metabolite transfer and their impact on water uptake

To better understand how electron flow influences the use of specific metabolic pathways by *D. chodatii* during autotrophic growth, a series of simulations were performed in which the ratios of LEF to CEF were fixed to values between 0.001 to 1,000 (Table 3). As the ratio of LEF to CEF reduces, the proton gradient across the chloroplast lumen steepens, which increases ATP generation. Increased levels of ATP fuel the biosynthesis of glyceraldehyde-3-phosphate (G3P) by phosphoglycerate kinase and G3P dehydrogenase in the chloroplast instead of the cytosol, and results in the regeneration of NADP in the chloroplast. Excess G3P is transferred from the chloroplast to the cytosol by a G3P-phosphate antiporter to fuel the pentose phosphate pathway in the cytosol and generate biomass precursors. The requirement for the generation of ATP in mitochondria will therefore drop, which in turn reduces the total flux through the TCA cycle (Table 3). The iDco_core model predicts a partial TCA cycle operating under reduced LEF to CEF ratios, where a cytosolic/mitochondrial oxaloacetate-isocitrate carrier transfers mitochondrial isocitrate to the cytosol and bypasses mitochondrial isocitrate dehydrogenase, 2-oxoglutarate dehydrogenase, and succinyl-CoA ligase. The cytosolic isocitrate dehydrogenase utilizes cytosolic isocitrate and regenerates cytosolic NADPH (Table 3). For all these simulations, the objective was to maximize biomass while keeping carbon dioxide uptake fixed. According to Table 3, as the ratio of LEF to CEF reduces, water uptake and oxygen evolution increase but maximum biomass remains unchanged. The possibility for cells to switch from linear to cyclic electron flow may be an adaptation of the photobiont to fluctuating water availability occurring during periods of desiccation. For the *E. pusillum–D. chodatii* lichen association, which colonizes soils in arid and semi-arid habitats, these fluctuations may occur daily (morning dew) or seasonally (rainy season). As predicted by the iDco_core model, different transporters are used for the translocation of key metabolites across different compartments to provide reducing power during the switch between different electron flow mechanisms. This suggests that environmental conditions lead to the differential expression of the genes encoding for these transporters.

**TABLE 3:** Fluxes (in mmol/gDCW/h) of select reactions predicted by the iDco_core model growing photoautotrophically at varying ratios of LEF to CEF

		Flux (mmol/gDCW.h)				
Reaction name	Reaction equation	LEF:CEF = 1,000	LEF:CEF = 10	LEF:CEF = 1	LEF:CEF = 0.1	LEF:CEF = 0.001
*D. chodatii* biomass	1.191 g6p[c] + 0.0 f6p[c] + 0.501 e4p[c] + 0.715 r5p[c] + 0.133 g3p[c] + 1.205 3 pg[c] + 1.002 pep[c] + 1.197 pyr[c] + 3.727 accoa[c] + 2.039 oaa[c] + 2.82 nad[c] + 49.06 nadph[c] + 53.35 atp[c] + 53.35 h2o[c] −> biomass[c] + 53.35 adp[c] + 53.35 h[c] + 53.35 pi[c] + 2.82 nadh[c] + 49.06 nadp[c] + 3.887 coa[c]	0.256765778	0.25676578	0.25676578	0.256765778	0.256765778
RuBisCO	co2[h] + h2o[h] + rb15 bp[h] −> 2.0 3 pg[h] + 2.0 h[h]	21.67595451	21.165293	18.3847303	14.59839173	13.52531814
Phosphoglycerate kinase, chloroplast	3 pg[h] + atp[h] <−> 13dpg[h] + adp[h]	0.362442872	2.03761054	11.1589344	23.89018021	27.05063628
Glyceraldehyde-3-phosphate dehydrogenase (NADP+) (phosphorylating)	13dpg[h] + h[h] + nadph[h] <−> g3 p[h] + nadp[h] + pi[h]	0.362442872	2.03761054	11.1589344	23.89018021	27.05063628
2-Oxoglutarate dehydrogenase	akg[m] + coa[m] + nad[m] + h[m] −> co2[m] + succoa[m] + nadh[m]	0.863918814	0.77610484	0.29795588	0	0
Isocitrate dehydrogenase (NAD)	icit[m] + nad[m] −> akg[m] + co2[m] + nadh[m]	3.747511291	3.5772908	2.65043657	0	0
Isocitrate dehydrogenase (NADP)	icit[c] + nadp[c] <−> akg[c] + co2[c] + nadph[c]	0	0	0	1.388323698	1.030632502
Succinyl-CoA ligase (ADP-forming)	atp[m] + coa[m] + h[m] + succ[m] <−> adp[m] + pi[m] + succoa[m]	−0.863918814	−0.7761048	−0.2979559	0	0
Carbon dioxide exchange	co2[e] <=>	−10	−10	−10	−10	−10
Proton exchange	h[e] <=>	−29.5398821	−26.843391	−12.160942	8.142980793	13.72920505
Water exchange	h2o[e] <=>	3.581629029	2.23338371	−5.1078409	−15.25980242	−18.05291455
Oxygen exchange	2[e] <=>	3.880627996	4.55475065	8.22536295	13.30134372	14.69789978
Photon exchange	photon[e] −>	−130.0882274	−130.03956	−129.77457	−129.972778	−129.7264752
Glyceraldehyde-3-phosphate dehydrogenase	g3p[c] + nad[c] + pi[c] <− 13dpg[c] + h[c] + nadh[c]	49.48434174	46.9580716	33.2024767	12.77234274	8.181121891
Phosphoglycerate kinase	3 pg[c] + atp[c] <−> 13dpg[c] + adp[c]	36.88741266	34.3611425	20.6055476	1.563737362	−3.385174688
ATP synthase, mitochondrial	2.67 h[c] + adp[m] + pi[m] −> atp[m] + 1.67 h[m] + h2o[m]	44.49737134	42.3115422	30.4096557	13.89207122	9.378920553
Cytochrome c oxidase, Complex IV	0.5 o2[m] + 4 h[m] + 2 cytcr[m] −> h2o[m] + 2 h[c] + 2 cytc[m]	13.92733756	13.2410481	9.50418753	4.477759467	3.019627703
NADH:ubiquinone oxidoreductase, Complex I	4 h[m] + nadh[m] + q8[m] −> nad[m] + q8 h2[m] + 3 h[c]	10.17982626	9.66375733	6.85375096	3.089435768	1.988995201
Cytochrome bc1:ubiquibol 8, Complex III	2 cytc[m] + 2 h[m] + q8 h2[m] −> 2 cytcr[m] + 4 h[c] + q8[m]	13.92733756	13.2410481	9.50418753	4.477759467	3.019627703
Succinate dehydrogenase (ubiquinone) complex II	q8[m] + succ[m] −> fum[m] + q8 h2[m]	3.747511291	3.5772908	2.65043657	1.388323698	1.030632502
Transketolase	e4p[c] + xu5 p__D[c] <−> f6 p[c] + g3 p[c]	−0.032159914	−0.0321599	−0.0321599	−0.032159914	−0.125366918
Ferredoxin-NADP+ reductase	2 nadp[h] + 4 fdxrd[h] −> 2 nadph[h] + 2 h[h] + 4 fdxox[h]	21.67776008	21.3346154	19.4661851	16.95297104	16.22390516
Photosystem II	2 h2o[t] + 4 photon[h] + 4 h[h] + 2 pq[t] −> o2[h] + 2 pqh2[t] + 4 h[t]	10.84429677	11.1752747	12.9774567	15.54022345	16.20771364
Cytochrome b6/f complex, linear electron flow	2 pqh2[t] + 4 pc[t] −> 2 pq[t] + 4 pch2[t] + 4 h[t]	10.83346331	10.1593407	6.48872836	1.412747587	0.016191522
Cytochrome b6/f complex, cyclic electron flow	2 pqh2[t] + 2 pc[t] + 2 h[h] −> 2 pq[t] + 2 pch2[t] + 6 h[t]	0.010833463	1.01593407	6.48872836	14.12747587	16.19152211
Photosystem I	4 photon[h] + 4 h[h] + 2 pch2[t] + 4 fdxox[h] −> 2 pc[t] + 4 fdxrd[h]	21.67776008	21.3346154	19.4661851	16.95297104	16.22390516
ATP synthase, chloroplast	adp[h] + 4.0 h[t] + pi[h] −> atp[h] + h2o[h] + 3.0 h[h]	21.69401028	22.8585165	29.1992776	38.14418484	40.51118833
Malate dehydrogenase	mal__L[c] + nad[c] <−> h[c] + nadh[c] + oaa[c]	45.87666977	43.4328062	30.1259165	10.65993955	6.426409895
Malate dehydrogenase (NADP+), chloroplast	h[h] + nadph[h] + oaa[h] −> mal__L[h] + nadp[h]	42.99307729	40.6316202	27.7734358	10.01576187	5.397174038
Pyruvate dehydrogenase, mitochondria	coa[m] + nad[m] + pyr[m] <−> accoa[m] + co2[m] + nadh[m]	4.704477346	4.53425685	3.60740263	2.345289754	1.987598557
3-Phospho-D-glycerate transport	3 pg[c] + pi[h] <−> pi[c] + 3 pg[h]	−42.98946614	−40.292976	−25.610526	−5.306603246	0
Glyceraldehyde 3-phosphate transport	g3p[c] + pi[h] <−> pi[c] + g3 p[h]	36.57961469	34.0533446	20.2977496	1.255939385	−3.819689656
Malate/oxaloacetate shuttle, chloroplast	mal__L[h] + oaa[c] −> mal__L[c] + oaa[h]	42.99307729	40.6316202	27.7734358	10.01576187	5.397174038
Dicarboxylate/tricarboxylate carrier (oaa:akg), mitochondrial	aa[c] + akg[m] <−> oaa[m] + akg[c]	2.883592477	2.80118596	2.35248069	0	0
Dicarboxylate/tricarboxylate carrier (oaa:icit), mitochondrial	aa[c] + icit[m] <−> oaa[m] + icit[c]	0	0	0	0.644177683	1.029235857
Phosphate transport, mitochondrial	h[c] + pi[c] <−> h[m] + pi[m]	45.36129015	43.087647	30.7076116	13.89207122	9.378920553

#### Sorbitol biosynthesis

**Fig 2:**
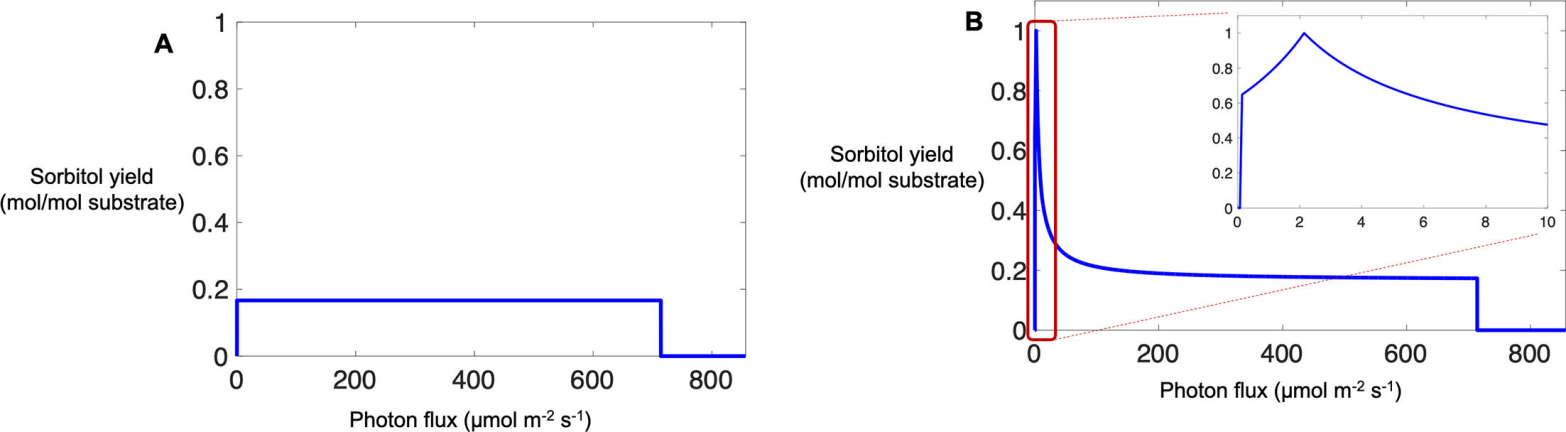
Sorbitol production yield (mol per mol substrate) at varying photon fluxes predicted by the iDco_core metabolic model of *D. chodatii* for growth with carbon dioxide (**A**), and glucose in addition to carbon dioxide (**B**) as the substrates. Glucose influx was fixed to 1 mmol/gDCW/h, while carbon dioxide uptake was left unconstrained. The inset (red box) shows expanded details.

For simulating sorbitol biosynthesis by the iDco_core model under autotrophic growth, the photon flux was progressively constrained to increasing values from zero to 850 µmol/m^2^/s while carbon dioxide uptake was left unconstrained. The objective was to maximize sorbitol flux. The maximum yield of sorbitol predicted by the model reached 0.17 mol per mol carbon dioxide for all photon fluxes up to a flux of 714 µmol/m^2^/s (Fig. 2A). Although the sorbitol yield remained constant, sorbitol exchange flux increased as photon flux increased.

Under mixotrophic growth, the yield of sorbitol reached a maximum of 1 mol per total moles of substrates (Fig. 2B). Sorbitol yield increased with increasing photon flux up to 2 µmol/m^2^/s and then decreased at higher photon fluxes (Fig. 2B) until reaching 0.17 mol per total moles of substrates. The predicted increase in sorbitol yield at extremely low light intensities reflects the heterotrophic growth of the alga, where glucose is utilized as the sole carbon source and carbon dioxide evolution closes carbon balance within the metabolic network. On the other extreme of high light intensities (photon fluxes of 400 µmol/m^2^/s and higher), the maximum sorbitol yield reaches its predicted maximum during autotrophic growth. Based on model predictions, it is theoretically feasible to obtain higher sorbitol production yields at photon fluxes above 2 µmol/m^2^/s if glucose is constantly fed into the system. For example, at a photon flux of 20 µmol/m^2^/s, the iDco_core model predicted that up to 48% more sorbitol can be produced, when the carbon dioxide to glucose ratio is 4.5:1, than when the photon flux is below 2 µmol/m^2^/s. In other words, higher sorbitol production yields can be obtained under mixotrophic conditions (with constant glucose input) than autotrophic conditions given a similar range of photon fluxes.

### Perspectives and future works

In this work, a metabolic flux modeling framework was developed for *Diplosphaera chodatii*, linking its genome to its metabolic fluxome. The model, iDco_core, establishes the computational basis for systems-level simulations and analyses of the metabolism of this alga. Importantly, this model enables the analysis of the metabolic capabilities of *D. chodatii* both as a free-living alga and as a partner in a symbiosis with the lichenized fungus *E. pusillum* (26). The model predicts aspects of both autotrophic and mixotrophic growth of the alga depending on light and nutrient availability, with growth predictions in agreement with published empirical observations of this alga. The iDco_core model of *D. chodati* supports the understanding that some algae employ both linear and cyclic electron flow mechanisms in their ETC, with cyclic electron flow being a common strategy among photosynthetic organisms to adapt to fluctuating environmental and metabolic conditions. We postulate that the adaptability of *D. chodatii* to fluctuating water levels might stem from its ability to switch on/off certain transporter proteins that assist in shuttling electrons via linear and/or cyclic electron flow mechanisms to meet specific ATP synthesis demands depending on water availability.

This work also provides insights into possible strategies for sorbitol production by *D. chodatii*. First, the predicted yield of sorbitol is higher during mixotrophic growth of the alga (i.e., carbon fixation along with constant glucose feeding), as compared to photoautotrophic growth with only carbon fixation. Second, during photoautotrophic growth at high photon fluxes, overflow metabolism may lead to increased starch biosynthesis, and consequently, increased sorbitol biosynthesis. The fact that *D. chodatii* uses light and atmospheric carbon dioxide for both its growth and synthesis of sorbitol suggests its relevance as an industrially attractive alternative to existing chemical and/or biochemical sorbitol production processes, which rely solely on organic carbon sources (13). However, the authors emphasize that all presented results suggest optimal theoretically feasible scenarios, which require additional evaluation before any final decisions can be devised. Further exploration of the *D. chodatii* metabolic model as a system for sorbitol production would require building enzyme kinetics and omics data specific to this species, to enable predicting more accurate fluxes. A major challenge universal across non-model eukaryotes is the low number of gene regions that can be functionally annotated to date with existing bioinformatic resources. For *D. chodatii* particularly, where published data on various biosynthesis and degradation pathways is scarce (e.g., lipids, fatty acids, polysaccharides, and even amino acids), developing fully “genome-scale” metabolic network reconstructions is extremely challenging. Additionally, the uncharacterized nature of cellular composition requires the iDco_core model to borrow data from non-phylogenetically related model microorganisms.

An important direction for future work would be the measurement of *D. chodatii* cellular composition and the analysis of key building blocks of lipids, fatty acids, and other macromolecules. Integration of these additional data would further increase the accuracy of predictions from the iDco_core model and improve our understanding of the metabolic interplay between this algal species and its lichenized fungal partner, more specifically in the context of *in vitro* synthesis of the lichen symbiosis. Finally, we highlight the need for further experimental evidence to fully elucidate the mechanisms underlying the observed growth patterns.

## SUPPLEMENTAL MATERIAL

Supplemental file captions spectrum.03660-23-s0001.docx Captions for Files S1 to S6.

Supplemental File S1 spectrum.03660-23-s0002.docx Amino acid sequences.

Supplemental File S2 spectrum.03660-23-s0003.docx Nucleotide sequences.

Supplemental File S3 spectrum.03660-23-s0004.xlsx Composition of biomass equations.

Supplemental File S4 spectrum.03660-23-s0005.xlsx iDco_core metabolic reconstruction.

Supplemental File S5 spectrum.03660-23-s0006.xlsx Functional annotations.

Supplemental File S6 spectrum.03660-23-s0007.xlsx Major transporter proteins.

ASM does not own the copyrights to Supplemental Material that may be linked to, or accessed through, an article. The authors have granted ASM a non-exclusive, world-wide license to publish the Supplemental Material files. Please contact the corresponding author directly for reuse.
