# Supplementary material for: Analyzing sorbitol biosynthesis using a metabolic network flux model of a lichenized strain of the green microalga Diplosphaera chodatii
Source: Microbiol Spectr. 2024 Dec 9;13(1):e03660-23. doi: 10.1128/spectrum.03660-23 (PMC11705836; doi:10.1128/spectrum.03660-23)
Supplement: Supplemental file captions — Captions for Files S1 to S6. [file spectrum.03660-23-s0001.docx]

**Supplementary files captions**

Supplementary File 1: Amino acid sequence file obtained from the structural annotations of the genome of *D. chodatii* CS-1475 (Gueidan et al 2023) and used here for functional annotation with OmicsBox.

Supplementary File 2. Nucleotide sequences corresponding to the amino acid sequence file obtained from the structural annotations of the genome of *D. chodatii* CS-1475 (Gueidan et al 2023) and used here for functional annotation with OmicsBox.

Supplementary File 3. Composition of biomass equations used in the core metabolic model of *D. chodatii.*

Supplementary File 4. Core metabolic model of *D. chodatii* containing reactions, metabolites, enzymes, metabolic pathways, and genes used to reconstruct the metabolic network.

Supplementary File 5. Functional annotations obtained from the protein sequences of *D. chodatii* CS-1475 (Gueidan et al 2023) using OmicsBox.

Supplementary File 6. Major proton/metabolite transporter proteins selected from the list of annotated proteins of *D. chodatii*.
