# Supplementary material for: Analyzing sorbitol biosynthesis using a metabolic network flux model of a lichenized strain of the green microalga Diplosphaera chodatii
Source: Microbiol Spectr. 2024 Dec 9;13(1):e03660-23. doi: 10.1128/spectrum.03660-23 (PMC11705836; doi:10.1128/spectrum.03660-23)
Supplement: Supplemental File S1 — Amino acid sequences. [file spectrum.03660-23-s0002.docx]

Link to the Supplementary File 1: Amino acid sequence file obtained from the structural annotations of the genome of *D. chodatii* CS-1475 (Gueidan et al 2023) and used here for functional annotation with OmicsBox. <https://figshare.com/s/53dd5a496ec782d907af>
